# OP76 Cost-Effectiveness Of Offering Human Papillomavirus Self-Sampling To Non-Attendees Of Organized Primary Cervical Cancer Screening In Germany

**DOI:** 10.1017/S0266462324001351

**Published:** 2025-01-07

**Authors:** Gaby Sroczynski, Lára R. Hallsson, Milena Muehler, Peter Hillemanns, Matthias Jentschke, Uwe Siebert

## Abstract

**Introduction:**

In Germany, organized cervical cancer screening with annual Papanicolaou (Pap) cytology for women age 20 to 34 years and three-yearly co-testing with human papillomavirus (HPV) and Pap for women as of age 35 years is standard. However, about 30 percent of women eligible for screening remain un/under-screened. We systematically evaluated benefits, risks, and cost-effectiveness of offering additional HPV self-sampling (HPV-SS) to non-attendees.

**Methods:**

A validated Markov model for the German context was used to evaluate different HPV-SS screening strategies compared to standard clinician-based screening: HPV-SS for non-attendees age 25 to 65, 30 to 65 or 35 to 65 years, every five years with regular invitation, either opt-in (invitation with link to order the test), or send-to-all (test sent with invitation). German clinical, epidemiological, and economic data (index year 2022/23), along with test accuracy and HPV-SS-attendance data from international meta-analyses and trials were incorporated. Outcomes included undiscounted life years gained (LYG) compared to standard screening without HPV-SS in non-attendees, and the incremental cost-effectiveness ratio (ICER; in EUR/LYG). Comprehensive sensitivity analyses were performed.

**Results:**

Incremental undiscounted effectiveness (compared to standard screening without HPV-SS) and discounted ICERs (compared to next effective) for non-dominated HPV-SS screening strategies were 0.00090 LYG (EUR22,700/LYG) for offering with five-yearly screening invitation an HPV-SS (opt-in) to non-attendees age 35 to 65, 0.00166 LYG (EUR25,900/LYG) for HPV-SS (send-to-all) age 35 to 65, 0.00167 (EUR726,000/LYG) for HPV-SS (send-to-all) age 30 to 65, and 0.00167 LYG (EUR1.78 million/LYG) for HPV-SS (send-to-all) age 25 to 65 years. Other opt-in strategies were dominated. Results were robust over a wide range of parameter variations.

**Conclusions:**

Offering HPV-SS (send-to-all) to non-attendees every five years as an additional strategy within the organized cervical cancer screening program is effective and cost effective in the German context. The results can be used to inform decision-makers and clinical guideline developers regarding incorporation of the specific version of HPV self-sampling into the established organized cervical cancer screening program in Germany.

